# Verbal threat learning does not spare loved ones

**DOI:** 10.1038/s41598-021-84921-3

**Published:** 2021-03-09

**Authors:** Cristina Morato, Pedro Guerra, Florian Bublatzky

**Affiliations:** 1 Department of Personality, Assessment, and Psychological Treatment, Faculty of Psychology, University of Granada, Granada, Spain; 2 Central Institute of Mental Health, Medical Faculty Mannheim/Heidelberg University, J5, 68159 Mannheim, Germany

**Keywords:** Motivation, Emotion, Learning and memory, Social neuroscience, Neuroscience, Psychology, Human behaviour

## Abstract

Significant others provide individuals with a sense of safety and security. However, the mechanisms that underlie attachment-induced safety are hardly understood. Recent research has shown beneficial effects when viewing pictures of the romantic partner, leading to reduced pain experience and defensive responding. Building upon this, we examined the inhibitory capacity of loved face pictures on fear learning in an instructed threat paradigm. Pictures of loved familiar or unknown individuals served as signals for either threat of electric shocks or safety, while a broad set of psychophysiological measures was recorded. We assumed that a long-term learning history of beneficial relations interferes with social threat learning. Nevertheless, results yielded a typical pattern of physiological defense activation towards threat cues, regardless of whether threat was signaled by an unknown or a loved face. These findings call into question the notion that pictures of loved individuals are shielded against becoming threat cues, with implications for attachment and trauma research.

## Introduction

Seeing your loved ones has particular benefits to human well-being and health. Going beyond the advantage of having a supportive social network, the presence of attachment figures has been shown to enhance life expectancy, physical health, and psychological resilience^1,2^. In addition, the mere vicarious presence of loved ones (e.g., by looking at pictures) is related to reduced pain and defensive behaviors^3–5^. However, attachment figures may also become a source of grief and misery, and recent translational research started examining the involved severe neurobiological and psychosocial deficits in humans and animals^6–10^.

As a highly social species, humans’ survival depends on the quality of their social network, and attachment figures provide a sense of safety and security. Looking at pictures of beloved faces evokes a variety of (emotional) memories and draws attention to certain situations that are difficult to ignore. On the psychophysiological level, a pattern of changes occurs that is distinctive of a positive emotional state^5,11,12^. This is shown, for instance, by a biphasic modulation of the heart rate (deceleration–acceleration), inhibition of defensive reflexes (e.g. startle reflex) and the corrugator muscle (frowning), and increases of zygomaticus muscle activity (smiling). In addition, activating a mental representation of attachment figures and supportive others has been shown to reduce pain experience^4,13,14^. For instance, the physical presence of the partner reduced pain, even without a need for interaction^3^. Similarly, Master et al.^15^ found that viewing a partner photograph and holding the partner’s hand while receiving thermal stimulations reduce pain perception more than holding an object or the hand of an unknown individual. Thus, viewing attachment figures or even their photograph is beneficial for coping with pain and stress, but little is known about social modulators of aversive learning.

As an experimental model to investigate affective learning, much research used experiential learning paradigms such as Pavlovian conditioning. In this procedure, a previously neutral stimulus (conditioned stimulus, CS) acquires an affective value by being paired with an appetitive or aversive event (e.g., electric shock serving as unconditioned stimulus, UCS). Importantly, this association leads to conditioned responses to the CS when it is presented alone, as reflected by enhanced autonomic arousal, primed defensive reflexive motor responses, and activation of a neural fear network (e.g., amygdala, anterior cingulate cortex)^16^. Some stimuli, which evolutionary threatened survival (e.g., snakes), have been proposed to be more readily conditioned as aversive, and such prepared fear associations are harder to extinguish^17,18^. Recent studies suggested a parallel notion of prepared safety stimuli, which evolutionary benefited survival and thus be more readily learned as safety cue inhibiting fear responses^19–21^. However, humans do not only learn by means of first-hand experiences but through observation and verbal instructions^22,23^. Despite their broad relevance for educational and clinical phenomena, for example, affective and expectancy learning, racial discrimination or phobias^24–26^, such social learning processes are still hardly understood.

In the present research, we examined the impact of verbal threat/safety learning while viewing loved and unknown faces serving as instructed cues for shock threat or safety. Moreover, instructional learning was used to reverse previously acquired threat and safety associations^27,28^, and to clarify whether these processes depend on stimulus relevance^29^. Previous studies have revealed that verbal threat instructions change psychophysiological responses to visual stimuli, even without having experienced the anticipated aversive events, leading to increased skin conductance, heightened corrugator electromyography activity, cardiac deceleration, and potentiated startle reflex^27,30,31^. The present study examined the capability of significant others in becoming threat or safety cues. Previous research showed that specific stimulus categories are more readily associated with aversive events, and more resistant to subsequent extinction learning (e.g., pictures of spiders or out-group members)^32^. An opposite pattern should be observed for stimuli that inherently signal safety—such as pictures of loved familiar people. Accordingly, inhibited fear acquisition and rapid extinction learning is expected for face pictures of loved relative to unknown people serving as instructed threat cues. This is assumed to result in less pronounced (or even non-significant) fear learning when loved faces cue threat (i.e., threat-potentiated startle response, enhanced SCR, initial HR-deceleration, and threat ratings). In contrast, pictures of unknown faces should more readily acquire aversive qualities when instructed as threat-cue in the second half of the experiment^28,33^.

## Methods

### Participants

Forty-five students (36 female, mean age = 20.04 years, *SD* = 1.93) were recruited from the University of Granada (Spain). Sample size was chosen similar to previous research using facial expressions and instructed threat manipulations^11,12,28,33,34^ and is in line with estimations based on G*power^35^. Statistical estimations indicate that N = 46 is required to detect instruction by face category interaction effects at a medium effect size (*f* = 0.20, power = 0.90, α error = 0.05, and assumed correlation of repeated measures = 0.5). Participants were in general good health with normal or corrected-to-normal vision. For some variables, data were lost because of recording errors with single sensors. However, no participant was completely excluded. For startle reflex and skin conductance, data from one participant were excluded in each case (final Ns = 44). As regards heart rate, two participants were removed from analyses (N = 43).

All participants were informed about the general experimental procedure and provided written informed consent prior to their participation. The ethics committee of the University of Granada (Spain) approved the experimental protocol, which complies with the APA ethical standards and the Declaration of Helsinki.

### Materials, design, and experimental presentation

Face photographs of four loved familiar (romantic partner, father, mother, best friend) and four unknown people (another participant's loved ones) were used. The selection of four loved identities was chosen based on previous research showing pronounced patterns of both central and peripheral responses (i.e., increased heart rate, zygomaticus muscle activity, SCRs, and P3/LPP components), that is distinctive of positive emotions and not attributable to familiarity or undifferentiated emotional arousal alone^5,11,12,34,36^. Moreover, with four identities per category, we were able to achieve a sufficient number of trials for our psychophysiological measurements (e.g., startle EMG) without excessive repetition of single face identities causing habituation effects. Finally, the used partial reversal design in the second experimental block requires at least four stimuli (i.e. maintain threat cue, maintain safety cue, reversed threat-to-safe cue, and reversed safe-to-threat cue; e.g.^27^). All face pictures were Caucasian, originated from Spain, and were matched for gender and age. For instance, if the participants own romantic partner was male, the corresponding picture of a friend had to be a female face (and vice versa). In addition, participants were asked to provide recent pictures of their mother and father. Picture materials were then matched for size (886 × 886 pixels), color (black and white), and background (light-colored).

In a first block, half of the pictures of each face category were instructed as signals for either threat of electric shocks (e.g., mothers and romantic partners) or safety (e.g. fathers and best friends). In a second block, instructed threat and safety associations were partially reversed, in that two faces of each category maintained their original meaning (e.g. loved/unknown mother signaling threat, and loved/unknown best friends signaling safety), and two other faces were reversed (e.g. now fathers cue threat-of-shock and romantic partners signal safety). The assignment of face identities to threat and safety condition was counterbalanced across participants. However, to reduce the impact of within-category variability on threat/safety learning (e.g., due to familiarity or age)^5^, we applied the restriction of having each one high- and one less-familiar person as threat/safety cue in each experimental block (see “Supplementary materials S1”).

Thus, the core experimental design (2 × 2 × 2) depicted Face Category (loved ones vs. unknown people), Cue (threat vs. safety) as repeated measures factors in the instantiation block, and in addition Contingency (maintained vs. reversed threat/safety) for the reversal block. In both blocks, threat and safety contingencies were verbally instructed and counterbalanced across participants. The sequence of stimulus presentation was pseudo-random with the restrictions that the same identity could not appear in more than two consecutive trials, and only three consecutive picture-startle or no-startle trials were presented in a row. Importantly, to focus on the impact of aversive anticipations (rather than experiences) no shocks were administered during the experiment. However, to enhance credibility of threat-of-shock instructions, a brief shock work-up procedure was carried out before the experiment started.

**Figure 1:**
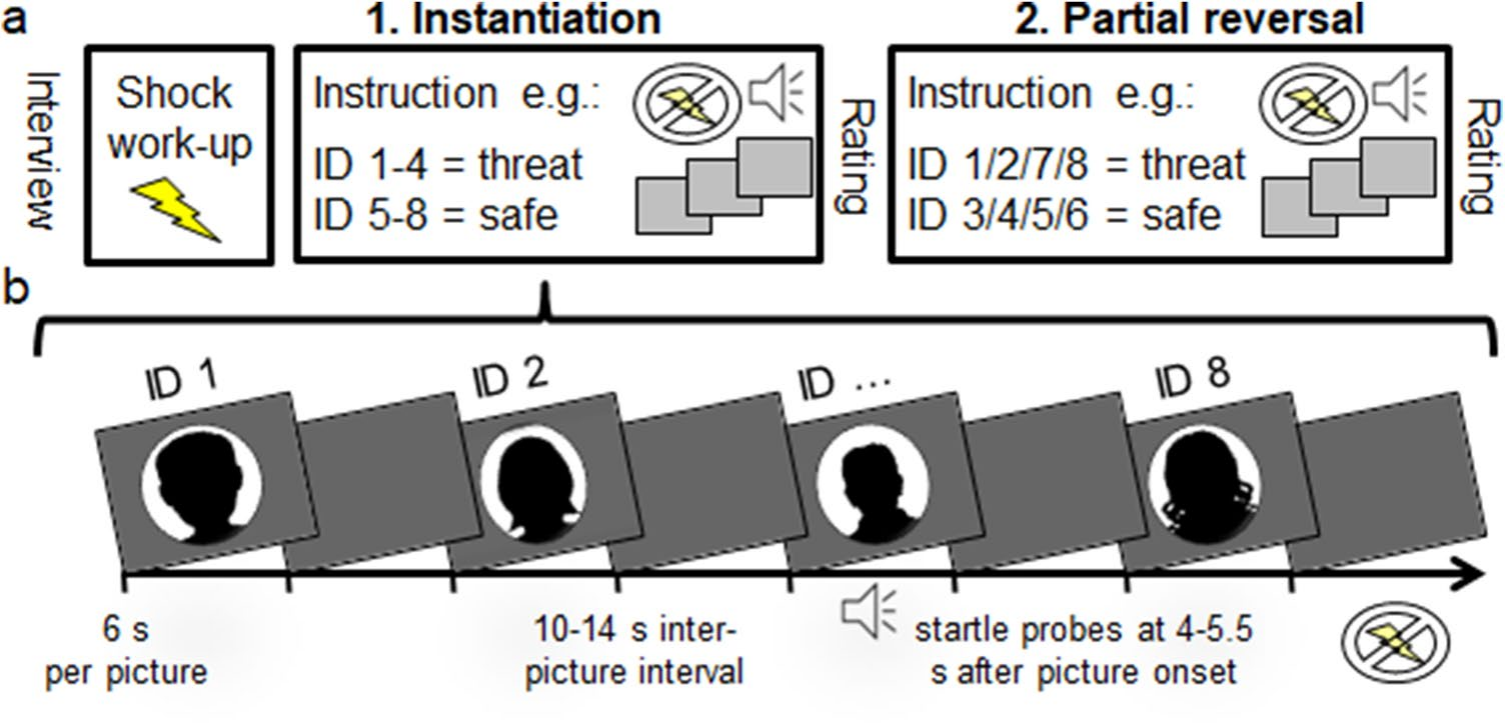
Schematic illustration of the experimental procedure. (**a**) An initial shock work-up procedure was carried out to ensure credibility of the threat-of-shock instructions. The first experimental block started with verbal instructions regarding which face identity (ID) is cueing threat or safety (instantiation). To this end, two loved and two unknown face identities were pointed out as cues for aversive shocks (e.g. both loved and unknown fathers and best friends), whereas the other four identities served as instructed safety cues (e.g. mothers and partners). In the partial reversal block, threat and safety associations were partially changed. Each one loved and unknown identity maintained cueing threat and safety, the associations of the other two identities were reversed. Note, the instructed contingencies between face identity and threat or safety were counterbalanced across participants. (**b**) For each block, all face identities were presented eight times (64 trials) and auditory startle probes were presented in half of the picture trials, four additional probes were presented during ITI. In order to focus on the impact of aversive anticipation (but not experience), no shocks were applied throughout the experiment.

The experiment began with a 2 min baseline period, followed by two blocks of 64 picture trials each, with every picture being presented 16 times throughout the experiment. Individual trials consisted of 4 s baseline period, 6 s picture presentation, a 4 s post-picture period and a varying inter-trial interval from 2 to 4 s (see Fig. 1). Pictures were presented at approximately 60 cm in front of the participants on a 19″ flat screen monitor. Auditory startle probes were delivered at either 4, 4.5, 5 or 5.5 s after picture onset in half of the picture trials (i.e. 32 probes per block) and were equally distributed across picture categories; four startle probes were also presented during the inter-trial intervals. Startle probes (105 dB, 50 ms) were produced by Coulbourn S81-02 noise generator, gated by a Coulbourn S82-24 audio-mixer amplifier (Coulbourn Instruments, Whitehall, PA) and presented through Telephonics TDH-49P earphones. Presentation software (Neurobehavioral Systems, Inc., Albany, CA) served to control stimulus presentation and VPM software^37^ to collect physiological measures. The electrical pulses were administered during the shock work-up procedure to the left forearm and generated by a Letica-shock-module LI 2700 (Letica, Barcelona, Spain).

### Procedure

An initial telephone interview served to clarify inclusion criteria: (1) having a highly positive relationship with their parents, romantic partner and best friend, (2) having a romantic relationship for at least 6 months up to 6 years (but not living together), and (3) having lived together with their parents at least until the age of 18 years. These latter criteria served to control for the duration of familiarity with regard to instructed threat/safety cues (i.e., parents are more familiar relative to romantic partner and best friend; for a discussion see^5^. Subsequently, instructions for preparation of picture materials were provided: frontal view of the face with a neutral expression, light-coloured background without objects behind, and the picture being taken by someone else other than the participant, to avoid background knowledge about the situational context of the picture.

Upon arrival in the laboratory, participants completed a picture familiarity rating to ensure that control pictures were unknown (if not, a different set of control faces was used), and scored relationship quality to their loved ones on a five-point Likert scale “How would you currently define your relationship with your father/mother/partner/friend on a scale ranging from 1 (very unsatisfactory) to 5 (very satisfactory)?” with 3 as a cut-off. Given the pre-selection and inclusion criteria, relationship quality with the romantic partner (*M* = 4.5, *SD* = 0.56), best friend (*M* = 4.24, *SD* = 0.54), mother (*M* = 4.42, *SD* = 0.64), and father (*M* = 4.39, *SD* = 0.64) was rated as very good. In addition, questionnaires on positive/negative affectivity (PANAS^38^; asking how much participants currently feel e.g., active, distressed) and general social support (MOS^39^; asking for e.g. the “availability of someone to help if confined to bed”) were completed. However, these questionnaire measures were not specifically related to the relationship with their loved ones and assessed for exploratory reasons only.

Subsequently, participants were seated in a sound-attenuated room, sensors were attached, and a shock work-up was carried out^40^. To this end, electrical stimulation was increased in steps of 0.1 mA until participants perceived stimuli (*M* = 0.28 mA, *SD* = 0.16) and reported shocks as “maximally unpleasant but not painful” (*M* = 1.34 mA, *SD* = 0.78). On average, 10.55 stimulations (*SD* = 6.55) were needed from the perceptual to the unpleasantness threshold. Key instructions were then given verbally about which face identities served as threat and safety cues (i.e. threat/safety contingencies) and the corresponding faces were shown on the instruction sheet. “If you see one of these four pictures, there is always a possibility of receiving an electric shock as long as the picture is present” (i.e. threat cues), while on the contrary “if you see any of these other four pictures, you will not receive any electric shock” (i.e. safety cues). In addition, the participants had the task of looking at all the pictures during the entire time they were on the screen. Following the first block, participants rated all faces regarding perceived threat.

Before the second block, threat and safety associations were partially reversed. Instructions were the same as for the initial instantiation of threat/safety contingencies but with the changed threat/safety pictures. By the end of the experiment, participants completed the Self-Assessment Manikin (SAM^41^) to rate all photographs as well as threat and safety conditions in terms of perceived valence, arousal, and dominance. After completing additional questionnaires on empathy and attachment style (Interpersonal Reactivity Index, IRI^42^; Experience of Close Relationship, ECR^43^), participants were debriefed and received course credits for participation.

### Data recording and reduction

To get a comprehensive picture of somatic and autonomic nervous system activation, we assessed a broad set of psychophysiological measures, which had been shown to be sensitive to threat instructions and pictorial stimuli (e.g.^30^). Skin conductance responses were recorded using Ag/AgCl electrodes with isotonic gel (Biopac Systems) placed on the hypothenar eminence of the left hand and was recorded using a Coulbourn V71-23 coupler module with a sampling rate of 50 Hz. The electrocardiogram was measured at lead II using two standard Ag/AgCl electrodes filled with hyper-conductive gel (Parker Laboratories, Inc, New Jersey, U.S.A.). A Coulbourn V75-04 bio-amplifier, connected to a V75-48 high performance band-pass filter, was used for signal conditioning. Frequencies below 1.5 and above 20 Hz were cancelled out and the electrocardiogram was acquired at 1000 Hz.

All EMG activity was recorded by means of miniature In Vivo Metrics electrodes filled with gel and separate Coulbourn V75-04 bioamplifiers. The raw signals were band-pass filtered (28–500 Hz) and subsequently rectified and integrated using a Coulbourn V75-24 integrator. Time constants and sampling rates were 500 ms and 20 ms for the zygomaticus and corrugator, as well as 100 and 1000 Hz for orbicularis muscles activity.

Startle responses were scored with an automated detection algorithm^44^, verified by visual inspection. The startle amplitude was defined as the difference between the peak and the onset of the response, in a time window between 20 and 120 ms after stimulus onset. To control for between-subject variability, startle amplitudes for each participant were transformed to *T*-scores.

Skin conductance responses, heart rate, zygomaticus, and corrugator activity were calculated by averaging across each half-second for the duration of the picture display and by subtracting the activity within 1 s prior to the picture onset.

### Data analysis

Data and syntax can be retrieved here: https://osf.io/fy2n7/?view_only=3c2abe24c3ee41fa84f613fecf1a70c0.

#### Self-report data

As a manipulation check, perceived threat was examined with a repeated measure ANOVA depicting the factors Cue (threat vs. safety), Face Category (loved vs. unknown), and Block (instantiation vs. reversal). Moreover, valence, arousal, and dominance ratings of the face pictures were analyzed by means of repeated measures ANOVAs including the within factors Cue (threat vs. safety) and Face Category (loved vs. unknown). Because these ratings were obtained only once at the end of the experiment, the factor Cue (threat vs. safety) could be tested only for those face pictures that maintained cueing threat or safety throughout the experiment. Finally, the credibility of threat/safety instructions during the instantiation and reversal block (asked during debriefing) was tested with a paired sample T-test.

#### Peripheral measures

For all peripheral measures, repeated-measures ANOVAs were calculated separately for each experimental block (instantiation and reversal) including the factors Faces Category (loved vs. unknown), Cue (threat vs. safety), and additionally Contingency (maintained vs. reversed) for the reversal block. The factor Time (12 half-seconds) was included to examine the temporal development of skin conductance, heart rate, zygomaticus, and corrugator EMG responses*.*

A significance level of *p* = 0.05 was used, partial eta square (η_p_^2^) was used as measure of effect size, and 95% confidence intervals are reported. Greenhouse–Geisser corrections were applied when necessary, and Bonferroni corrections were applied for post-hoc analyses.

## Results

### Self-report data

**Figure 2:**
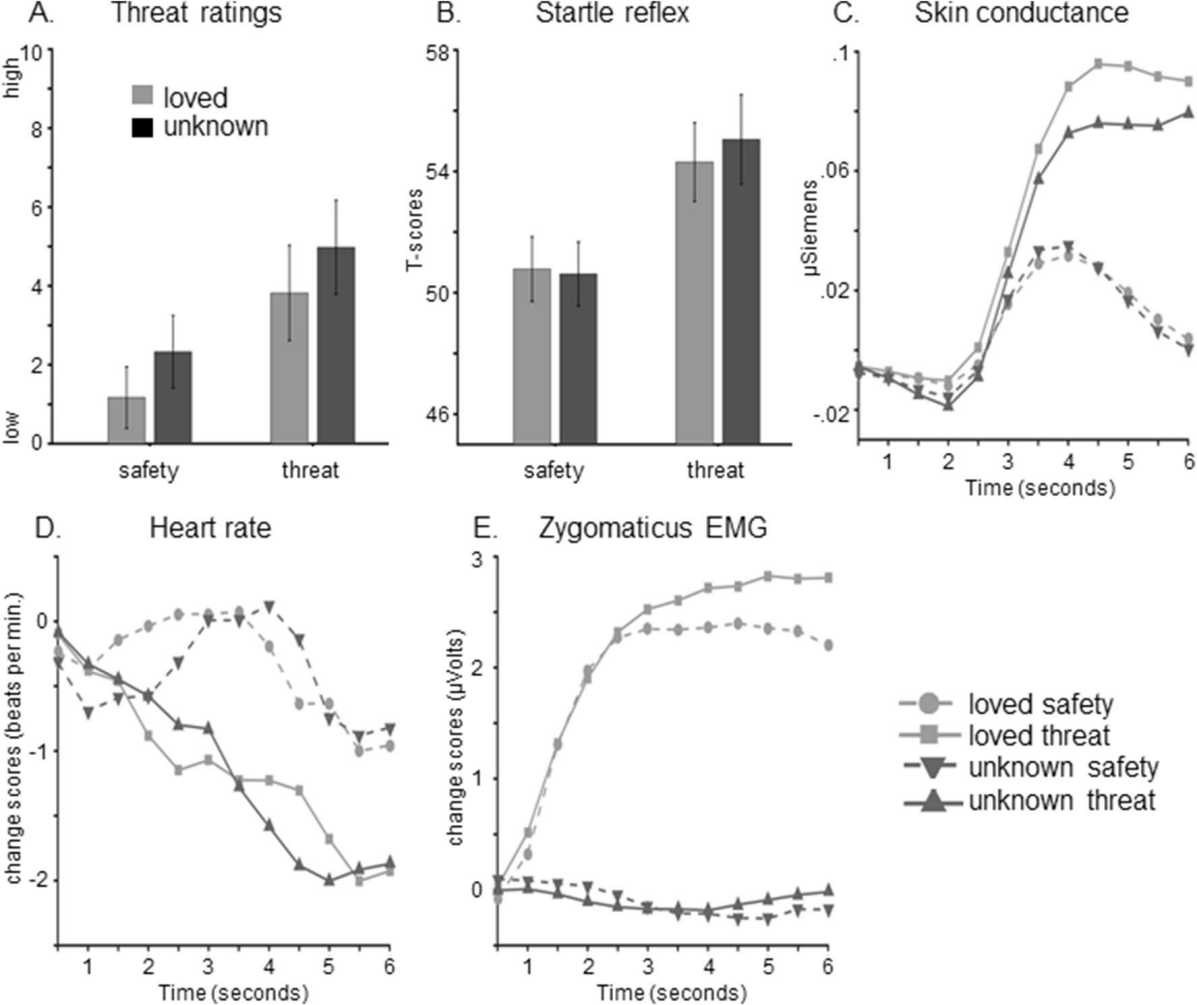
Instantiation of threat and safety contingencies (instantiation block). (**A**) Threat ratings, (**B**) eye-blink startle reflex, (**C**) skin conductance responses, (**D**) heart rate changes, and (**E**) zygomaticus activity as a function of Cue (threat, safety) and Face Category (loved, unknown).

**Figure 3:**
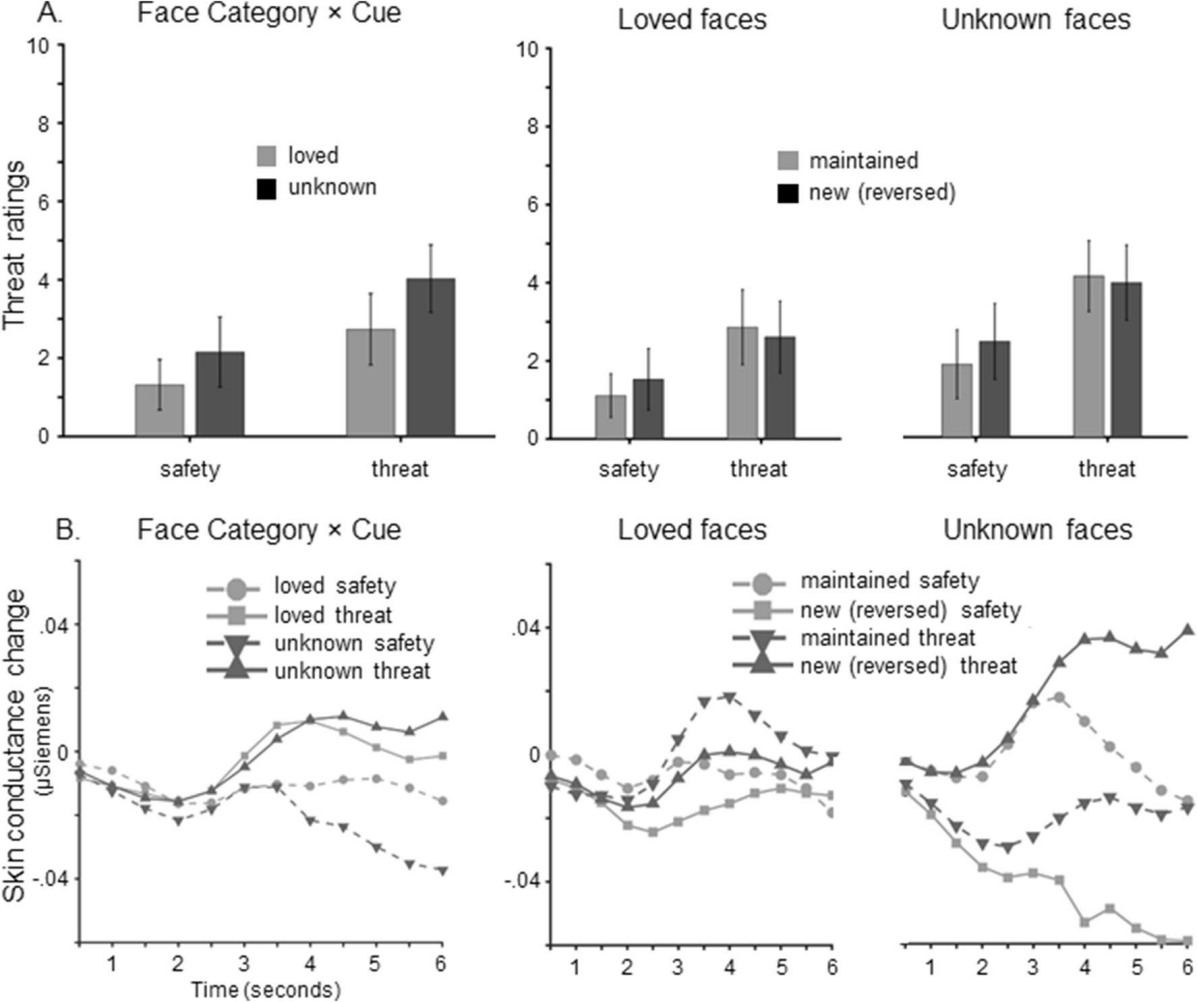
Partial reversal of instructed threat and safety contingencies (reversal block). (**A**) Threat ratings, and (**B**) skin conductance responses as a function of Instruction (threat, safety) and Face Category (loved, unknown). Separate graphs show ratings and SCR for loved faces (middle column) and unknown faces (right side) to illustrate the interaction with Contingency (maintained, reversed).

The perceived threat was rated after both instantiation and reversal block (see Figs. 2A and 3A). As predicted, instructed threat cues were more threatening than safety cues in the instantiation block, Cue *F*(1,35) = 23.22, *p* < 0.001, η_p_^2^ = 0.40, and unknown faces more threatening than loved faces, Face Category *F*(1,35) = 34.01, *p* < 0.001, η_p_^2^ = 0.49. No interaction emerged for the instantiation block, Cue × Face Category *F*(1,35) = 0.0,* p* = 1.0, η_p_^2^ = 0.0.

Similarly, for the reversal block, threat cues and unknown faces were perceived as more threatening relative to safety cues and loved faces, Cue *F*(1,35) = 29.42, *p* < 0.001, η_p_^2^ = 0.46, and Face Category *F*(1,35) = 33.71, *p* < 0.001, η_p_^2^ = 0.49. Interestingly, however, threat ratings revealed a significant interaction Cue × Face Category, *F*(1,35) = 4.75, *p* < 0.05, η_p_^2^ = 0.12. After reversal learning, all threat cues were perceived as more threatening than safety cues regardless of face category, all *ps* < 0.001, but this threat effect was more pronounced for unknown compared to loved people.

At the end of the experiment, the pictures were rated once in terms of valence, arousal and dominance (see Table 1). For valence ratings, loved faces were more pleasant relative to unknown faces, Face Category *F*(1,41) = 136.65, *p* < 0.001, η_p_^2^ = 0.77, but neither the main effect Cue, *F*(1,41) = 1.65, *p* = 0.21, η_p_^2^ = 0.039, nor the interaction Cue × Face Category was significant, *F*(1,41) = 0.06, *p* = 0.82, η_p_^2^ < 0.01. Self-reported arousal did not differ between loved and unknown faces, Face Category *F*(1,41) = 0.45, *p* = 0.51, η_p_^2^ = 0.01, but was more pronounced for threat relative to safety cues, Cue *F*(1,41) = 4.50, *p* = 0.04, η_p_^2^ = 0.10. No interaction Cue × Face Category was observed, *F*(1,41) = 0.16, *p* = 0.69, η_p_^2^ < 0.01. Dominance ratings showed neither main nor interaction effects, Cue *F*(1,41) = 1.01, *p* = 0.32, η_p_^2^ = 0.02, Face Category *F*(1,41) = 1.97, *p* = 0.17, η_p_^2^ = 0.05, and Cue × Face Category *F*(1,41) = 0.10, *p* = 0.76, η_p_^2^ < 0.01.

**Table 1:** Ratings of picture valence, arousal, dominance, and perceived threat as a function of Face Category (loved vs. unknown) and Cue (threat vs. safety).

Category	Cue	Valence			Arousal			Dominance			Threat		
		*M*	*SD*	95% CI	*M*	*SD*	95% CI	*M*	*SD*	95% CI	*M*	*SD*	95% CI
Loved	Safety	7.93	0.21	[7.50, 8.36]	4.43	0.39	[3.64, 5.21]	5.38	0.25	[4.87, 5.89]	1.36	0.32	[0.71, 2.01]
	Threat	7.69	0.23	[7.23, 8.16]	4.98	0.34	[4.30, 5.66]	5.12	0.20	[4.72, 5.52]	3.22	0.46	[2.28, 4.16]
Unknown	Safety	4.83	0.19	[4.45, 5.22]	4.12	0.25	[3.62, 4.62]	5.14	0.25	[4.65, 5.64]	2.40	0.41	[1.56, 3.23]
	Threat	4.52	0.23	[4.06, 4.99]	4.86	0.32	[4.21, 5.50]	4.76	0.26	[4.24, 5.28]	4.54	0.45	[3.62, 5.45]

Note, threat ratings are merged across blocks.

Finally, in the debriefing interview, participants rated the threat instruction in the first block as more credible than in the second block, *t* = 9.13, *p* < 0.001 (instantiation block: *M* = 9.12, *SD* = 1.25; reversal block: *M* = 5.59, *SD* = 2.43).

### Startle reflex

For the instantiation block, the startle reflex was potentiated when viewing instructed threat relative to safety cues, Cue *F*(1,43) = 39.05, *p* < 0.001, η_p_^2^ = 0.48 (see Fig. 2B and Table 2). Interestingly, no difference was observed between loved and unknown faces, Face Category *F*(1,43) = 0.16, *p* = 0.69, η_p_^2^ < 0.01, and no interaction emerged for Cue × Face Category *F*(1,43) = 0.52, *p* = 0.48, η_p_^2^ = 0.01, thus, indicating threat-potentiated startle reflex regardless of whether loved or unknown faces cued threat.

**Table 2:** Defensive reactions as a function of Block (instantiation vs. reversal), Face Category (loved vs. unknown) and Instruction (threat vs. safety) and Contingency (maintained vs. reversed).

Block	Category	Contingency	Startle			SCR			HR			Zygomaticus			Corrugator		
			*M*	*SD*	95% CI	*M*	*SD*	95% CI	*M*	*SD*	95% CI	*M*	*SD*	95% CI	*M*	*SD*	95% CI
Block 1	Loved	**Safety**–Safety	50.79	3.72	[49.65, 51.92]	0.01	0.05	[− 0.00, 0.03]	− 0.24	2.14	[− 0.89, 0− .42]	1.87	5.09	[0.34, 3.40]	− 0.21	0.57	[− 0.38, − 0.04]
		**Threat**–Threat	53.61	6.35	[51.68, 55.54]	0.06	0.14	[0.01, 0.10]	− 0.91	2.50	[− 1.68, − 0.14]	2.63	6.77	[0.60, 4.67]	− 0.10	0.52	[− 0.25, 0.06]
		**Safety**–Threat	50.77	5.21	[49.19, 52.36]	0	0.06	[− 0.01, 0.02]	− 0.43	1.84	[− 1.00, − 0.13]	1.82	4.29	[0.53, 3.11]	− 0.24	0.68	[− 0.44, − 0.03]
		**Threat**–Safety	55	5.07	[53.46, 56.54]	0.03	0.09	[0.00, 0.06]	− 1.32	2.00	[− 1.94, − 0.71]	1.55	3.41	[0.52, 2.57]	− 0.10	0.52	[− 0.26, 0.05]
	Unknown	**Safety**–Safety	50.25	4.80	[48.79, 51.71]	0	0.07	[− 0.02, 0.02]	− 0.49	1.82	[− 1.05, 0.06]	0.05	1.03	[− 0.25, 0.36]	0.26	0.38	[0.14, 0.37]
		**Threat**–Threat	54.8	6.12	[52.94, 56.66]	0.04	0.11	[0.00, 0.07]	− 0.91	2.22	[− 1.60, − 0.23]	− 0.12	1.23	[− 0.49, 0.25]	0.44	0.57	[0.27, 0.61]
		**Safety**–Threat	50.98	4.84	[49.51, 52.45]	0.01	0.05	[− 0.00, 0.03]	− 0.33	1.90	[− 0.91, − 0.26]	− 0.26	0.93	[− 0.54, 0.02]	0.22	0.33	[0.12, 0.32]
		**Threat**–Safety	55.31	6.98	[53.18, 57.43]	0.03	0.09	[0.00, 0.06]	− 1.35	2.03	[− 1.98, − 0.73]	0.07	0.55	[0.24, 0.10]	0.035	0.45	[.022, 0.49]
Block 2	Loved	Safety–**Safety**	46.2	3.36	[45.18, 47.23]	− 0.01	0.08	[− 0.03, 0.02]	− 0.11	2.01	[− 0.73, − 0.50]	0.76	3.07	[− 0.16, 1.69]	− 0.04	1.03	[− 0.35, 0.27]
		Threat–**Threat**	48.75	4.65	[47.33, 50.16]	0	0.05	[− 0.02, 0.02]	− 0.76	2.18	[− 1.43, − 0.09]	0.89	2.72	[0.08, 1.71]	0.08	0.70	[− 0.12, 0.30]
		Safety–**Threat**	48.06	4.57	[46.67, 49.45]	[− 0.01	0.07	[− 0.04, 0.01]	− 0.24	2.19	[− 0.91, − 0.43]	1.10	4.27	[− 0.18, 2.38]	0.01	0.70	[− 0.20, 0.22]
		Threat–**Safety**	46.97	3.58	[45.88, 48.06]	[− 0.01	0.10	[− 0.04, 0.02]	− 0.43	2.11	[− 1.08, − 0.21]	1.43	4.88	[− 0.03, 2.90]	0.10	0.82	[− 0.14, 0.35]
	Unknown	Safety–**Safety**	45.6	3.06	[44.66, 46.53]	[− 0.00	0.05	[− 0.02, 0.02]	− 0.04	2.14	[− 0.70, 0.61]	− 0.05	0.92	[− 0.33, 0.22]	0.24	0.45	[0.11, 0.38]
		Threat–**Threat**	47.22	3.98	[46.01, 48.43]	[− 0.02	0.08	[− 0.04, 0.00]	− 0.33	2.17	[− 1.00, − 0.34]	0.01	1.10	[− 0.32, 0.34]	0.34	0.40	[0.22, 0.46]
		Safety–**Threat**	49.11	5.06	[47.57, 50.65]	[− 0.04	0.07	[− 0.06, − 0.02]	− 0.05	1.81	[− 0.61, − 0.50]	− 0.28	1.21	[− 0.65, 0.08]	0.33	0.50	[0.18, 0.48]
		Threat–**Safety**	46.58	3.33	[45.57, 47.60]	0.02	0.10	[− 0.01, 0.05]	− 0.28	2.16	[− 0.95, 0.38]	− 0.16	0.77	[− 0.39, 0.07]	0.32	0.37	[0.21, 0.43]

The actual instruction (threat or safety) for each block is written bold.

After reversal instructions, startle reflex was potentiated for threat compared to safety cues, Cue *F*(1,43) = 13.69, *p* < 0.001, η_p_^2^ = 0.24. No differences were observed between cues that maintained or reversed their meaning, Contingency *F*(1,43) = 2.61, *p* = 0.11, η_p_^2^ = 0.06, or between loved and unknown faces, Face Category *F*(1,43) = 0.99, *p* = 0.326, η_p_^2^ = 0.02. Although not significant, the only evidence of a modulating influence of face category emerged for the interaction Face Category × Contingency, *F*(1,43) = 3.54, *p* = 0.067, η_p_^2^ = 0.076, which showed a more pronounced startle reflex for reversed compared to maintained unknown faces, *p* = 0.02, but not for loved faces, *p* = 0.95. Neither Cue × Face Category nor Cue × Contingency × Face Category reached significance, *Fs*(1,43) = 0.12 and 1.51, *ps* = 0.74 and 0.23, η_p_^2^ < 0.01 and = 0.03.

### Skin conductance responses

Skin conductance responses evolved over Time, *F*(11,473) = 21.22, *p* < 0.001, η_p_^2^ = 0.33, during the instantiation block. No differences were observed between loved and unknown faces, Face Category *F*(1,43) = 0.62, *p* = 0.44, η_p_^2^ = 0.01, but SCRs were enhanced for threat relative to safety cues, Cue *F*(1,43) = 7.81, *p* = 0.008, η_p_^2^ = 0.15, and this effect varied across time, Cue × Time *F*(11,473) = 8.96, *p* = 0.003, η_p_^2^ = 0.17 (see Figs. 2C and 3B, Table 2). Planned comparisons revealed these threat effects significant between time points 3.5–6 s after picture onset (all *ps* < 0.026). Moreover, the non-significant interaction Cue × Face Category, *F*(1,43) = 0.29, *p* = 0.60, η_p_^2^ = 0.01, indicates that loved and unknown faces served equally well as threat and safety cues during the instantiation block.

In the reversal block, SCRs did not vary over Time, *F*(11,473) = 1.32, *p* = 0.27, η_p_^2^ = 0.03, Cue *F*(1,43) = 2.66, *p* = 0.11, η_p_^2^ = 0.06, Contingency *F*(1,43) = 0.49, *p* = 0.49, η_p_^2^ = 0.01, or for Face Category *F*(1,43) = 0.27, *p* = 0.61, η_p_^2^ = 0.01. Importantly, however, a significant interaction Cue × Contingency emerged, *F*(1,43) = 4.57, *p* = 0.038, η_p_^2^ = 0.096. Planned comparisons confirmed that reversed threat cues (previously safe) resulted in increased SCRs compared to the reversed safety condition (previously threatening), *p* = 0.02, and reversed elicited lower responses compared to maintained safety cues, *p* = 0.015. Moreover, the instructed threat effects tended to vary across time, Cue × Time *F*(11,473) = 2.85, *p* < 0.079, η_p_^2^ = 0.06 (Fig. 2), and a marginal interaction Face Category × Cue × Contingency was observed, *Fs*(1,43) = 3.99, *p* = 0.052, η_p_^2^ = 0.09. Follow-up analyses indicate that SCRs were more pronounced to unknown faces that were newly learned as cues for threat relative to safety, *p* = 0.004. This was not observed for unknown faces which maintained cueing threat/safety, *p* = 0.213, and no differences emerged for loved faces, all *ps* > 0.663. While no further two- or three-way interaction approached significance, *F*s < 1.23, *p* > 0.30, η_p_^2^ < 0.03, however, the overall four-way interaction Cue × Contingency × Face Category × Time was significant, *F*(11,473) = 3.82, *p* < 0.023, η_p_^2^ = 0.08, indicating that instructed threat and reversal effects evolved over time specifically for unknown face pictures (Fig. 3B).

### Phasic heart rate changes

In the instantiation block, heart rate decreased over Time, *F*(11,462) = 10.54, *p* < 0.001, η_p_^2^ = 0.20, and for threat compared to safety cues, Cue *F*(1,42) = 10.03, *p* = 0.003, η_p_^2^ = 0.19. No main effects of Face Category or Contingency were observed, Cue *F*(1,42) = 0.04 and 1.13, *p* = 0.84 and 0.30, η_p_^2^ < 0.01 and = 0.03. Importantly, an interaction Cue × Time, *F*(11,462) = 10.96, *p* < 0.001, η_p_^2^ = 0.21, indicates that viewing safety cues provoked a biphasic pattern of heart rate changes (deceleration-acceleration, see Fig. 2D, Table 2). In contrast, threat cues were associated with a sustained deceleration, starting at 2.5 s after picture onset, and lasting for the entire presentation period, *ps* < 0.009. Neither the interaction Cue × Face Category, *F*(1,42) = 0.03, *p* = 0.86, η_p_^2^ < 0.01, nor any other higher-order interaction approached significance, *Fs* < 1.07, *ps* > 0.31, η_p_^2^ < 0.03.

In the reversal block, phasic heart rate showed a decrease over Time, *F*(11,462) = 6.12, *p* = 0.002, η_p_^2^ = 0.13, and a marginal main effect Cue, *F*(1,42) = 2.92, *p* = 0.095, η_p_^2^ = 0.07, which indicates more deceleration for threat compared to safety cues. No significant differences were observed for Face Category and Contingency, *Fs*(1,42) = 0.71 and 0.08, *ps* = 0.404 and 0.78, η_p_^2^ = 0.01 and < 0.01. The only significant interaction effect during the reversal block emerged for Cue × Time, *F*(11,462) = 5.06, *p* = 0.002, η_p_^2^ = 0.11, indicating that threat compared to safety cues elicited a deceleration, irrespective of whether they were maintained or reversed, loved or unknown faces. These threat effects started at 4 s after picture onset and were significant for the remaining presentation period (all *ps* < 0.047). No further main or interaction effect reached significance, *Fs* < 2.92, *p* > 0.10, η_p_^2^ < 0.07.

### Zygomaticus EMG

Overall, the zygomaticus EMG activity increased over Time, *F*(11,484) = 6.03, *p* = 0.01, η_p_^2^ = 0.12, and was significantly enhanced when loved faces were viewed compared to unknown faces during the instantiation block, Face Category *F*(1,44) = 8.90, *p* = 0.005, η_p_^2^ = 0.17 (see Fig. 2E, Table 2). No main effects were observed for Cue or Contingency, *Fs*(1,44) = 0.74 and 1.34, *ps* = 0.40 and 0.25, η_p_^2^ = 0.02 and 0.03. A significant interaction Face Category × Time was found, *F*(11,484) = 8.32, *p* = 0.003, η_p_^2^ = 0.16, indicating enhanced zygomaticus activity for loved compared to unknown faces starting from 1 s after picture onset to the end of presentation, all *ps* < 0.01. Neither Cue × Time, *F*(11,484) = 2.19, *p* = 0.10, η_p_^2^ = 0.05, Cue × Category, *F*(1,44) = 0.78, *p* = 0.38, η_p_^2^ = 0.02, nor any other interaction reached significance during the instantiation block, *Fs* < 2.28, *ps* > 0.14, η_p_^2^ < 0.05.

In the reversal block, participants tended to smile more when they saw a loved compared to an unknown faces, Face Category *F*(1,44) = 4.05, *p* = 0.05, η_p_^2^ = 0.08. No other main effect reached significance, Time *F*(11,484) = 3.06, *p* = 0.08, η_p_^2^ = 0.07, Cue *F*(1,44) = 1.80, *p* = 0.19, η_p_^2^ = 0.04, Contingency *F*(1,44) = 0.41, *p* = 0.53, η_p_^2^ = 0.01. The interaction Face Category × Time and Face Category × Contingency also failed to reach significance, *Fs* = 3.33 and 3.63, *ps* = 0.069 and 0.063, η_p_^2^ = 0.07 and 0.08. No other main or interaction effects were found, *Fs* < 0.71, *ps* > 0.52, η_p_^2^ < 0.02.

### Corrugator EMG

In the instantiation block, enhanced corrugator activity was observed for threat relative to safety cues, Cue *F*(1,44) = 10.68, *p* = 0.002, η_p_^2^ = 0.20, and unknown compared to loved faces, Face Category *F*(1,44) = 33.89, *p* < 0.001, η_p_^2^ = 0.44 (see Table 2). Although the main effect Time missed significance, *F*(11,484) = 2.92, *p* = 0.06, η_p_^2^ = 0.06, threat effects evolved over time, Cue × Time *F*(11,484) = 6.26, *p* = 0.003, η_p_^2^ = 0.12, with threat enhanced activity after 1.5 s following picture onset, all *ps* < 0.014. Moreover, the interaction Face Category × Time was significant, *F*(11,484) = 28.12, *p* < 0.001, η_p_^2^ = 0.39, indicating enhanced activity for unknown compared to loved faces after 1 s of picture presentation until 6 s, all *ps* < 0.001. The interaction Cue × Face Category × Time was not significant, *F*(11,484) = 1.83, *p* = 0.16, η_p_^2^ = 0.04.

Similarly, during the reversal block, more activity was found for threat relative to safety cues, *F*(1,44) = 5.46, *p* = 0.024, η_p_^2^ = 0.11, and unknown faces compared to loved faces, Face Category *F*(1,44) = 6.92, *p* = 0.012, η_p_^2^ = 0.14. Moreover, corrugator activity varied as a function of Time, *F*(11,484) = 5.91, *p* = 0.004, η_p_^2^ = 0.12, and Face Category × Time, *F*(11,484) = 8.37, *p* = 0.003, η_p_^2^ = 0.16, showing enhanced activity toward unknown faces starting from 1.5 to 6 s, all *ps* < 0.039. Corrugator activity showed no more significant main or interaction effect, *Fs* < 0.85, *ps* > 0.362, η_p_^2^ < 0.02.

## Discussion

The present study examined whether pictures of significant others—the romantic partner, parents, or best friends—are more resistant to becoming threat cues than pictures of unknown people^20^. We further predicted that unknown faces would more readily acquire aversive qualities when threat-associations were reversed. A broad set of psychophysiological measures showed pronounced defensive responding towards face identities, which served as instructed threat relative to safety cues. This differential response pattern emerged for measures of the somatic nervous system (threat-potentiated startle reflex and corrugator EMG), the autonomous nervous system (enhanced SCRs and heart rate deceleration), as well as for self-report (threat and arousal ratings). Interestingly, the zygomaticus muscle was the only measure insensitive to threat instructions. Participants smiled more when viewing their loved ones, regardless of whether they cued threat or safety. Importantly, for the instantiation of threat-associations, no interaction effects were observed between face category and threat/safety instructions for none of the dependent variables. Thus, pictures of loved people became threat cues as easily as it was observed for pictures of unknown people. Regarding reversal learning, however, some indications suggest that changing safety to threat worked better with unknown faces. Taken together, no evidence was found that pictures of loved familiar faces were resistant against becoming threat cues, but unknown faces may be more easily learned as new threat cues.

Learning about potential threats by means of social communication is highly beneficial, because an individual does not need to undergo aversive experiences him or herself^22,45^. This notion has received much support by research showing that the mere verbal instruction about the occurrence of threats is sufficient to provoke a pronounced psychophysiological pattern of defensive responding^30,31,46^. The present study replicates these findings within the domain of face and person perception. When viewing face identities that were associated with shock threat (relative to safety), participants were more aroused (enhanced SCRs and arousal rating), oriented towards the threat cue (heart rate deceleration), and defensive reflex activity was potentiated (startle reflex). Moreover, participants tended to frown more towards threat relative to safe identities (enhanced activity of the corrugator muscle). Thus, the mere verbal statement that a person might be dangerous primed defensive psychophysiological responding when viewing these individuals.

Knowledge about other people, however, is malleable and can be flexibly updated based on new information. Verbal instructions are particularly effective in changing affective associations^47–50^. For instance, Costa et al.^27^ showed that neutral stimuli associated with threat-of-shock or safety can be reversed from cueing threat to safety and vice versa. Similarly, verbal threat instantiation and reversal instructions can readily override the implicit affective meaning of emotional facial expressions (e.g. a smile may also signal threat^28,33^). Importantly, however, reversal learning implicates the workings of (at least) two concurrent processes: the inhibition of previously learned threat-associations, while a new threat-association is established^51^. As indicated by self-reported threat (and, on an exploratory basis, for startle reflex and SCR^33^), the present data provide some indication for the notion that new threat-associations are more readily acquired when threat is linked to unknown people, while concurrently loved people become new safety cues.

While encounters with the ‘unknown’ may be more likely to involve a risk of danger, on the contrary, social relationships with romantic partners and good friends are important health factors^2,12,52^. Here, recent conditioning research suggested significant others as prepared safety cues^19,21^. For instance, using a fear conditioning procedure with pictures of supportive others, unknown people, and neutral objects as conditioned stimuli (100% reinforcement schedule), the authors reported no differential fear conditioning, as measured by skin conductance responses, towards social-support figures serving as CS+ compared to CS−^20^. The present data do not support this notion. During instantiation, we did not find differential threat/safety learning towards pictures of loved compared to unknown face pictures, for none of the psychophysiological response measures (ratings, startle EMG, SCR, heart rate, and facial EMG). Moreover, for reversal learning, threat rating and SCR data point to the notion that unknown people may act as prepared fear stimuli relative to loved ones. While several methodological differences may explain the divergent findings (e.g., dependent variables, number of trials, selection of stimuli^3,53^), several theoretical aspects are of particular interest to further our understanding of the social factors involved in associative threat and safety learning.

First, we employed instructional learning, which establishes an association between a particular face identity and UCS by means of verbal instructions but not own experiences. Thus, threat learning occurs with a 0% reinforcement rate and, accordingly, the absence of shocks during the experiment does not necessarily lead to quick extinction learning, as it usually occurs in classical conditioning designs (depending on reinforcement schedule). Such instructed threat associations have been shown to persist within and even across repeated test days without experiencing the aversive events^31,54^, reflecting the workings of worries and apprehensions in anticipatory anxiety. On the other side, instructions can critically shape the impact of previous learning history of allegedly threatening or safe persons^49^. For instance, instructed information has been shown to change feedback-driven aversive learning and still little is known about the combined effects of different learning pathways and prior knowledge (e.g.^48,55^). Focusing on the neurobiological mechanisms involved in the social acquisition, maintenance and extinction of rather cognitive aspects of fear and anxiety may be particular informative.

Second, the use of pictures displaying loved people may interfere less with threat learning compared to pictures of supportive-others. In the present study, we selected participants solely based on their reported high relationship quality but not on perceived social support. Thus, even attachment figures with whom perceived relationship quality is very high, do not necessarily imply helpful support in a threatening situation. Here, the physical presence or absence as well as the type of prosocial or helping behavior might be a more relevant factor than the person offering support^13,15,56,57^. For instance, holding hands with a loved one reduces reported unpleasantness during the anticipation of shocks relative to no hand holding (in happily married women^56^) or holding hands with a stranger^58^. Moreover, this social regulatory process was associated with inhibition of a threat-related neural network (involving lateral prefrontal, cingulate, as well as posterior parietal cortices), which has been associated with salience detection, vigilance, and emotion regulation (e.g.^58,59^). Following on from this, the direct comparison of more or less familiar or supportive individuals (e.g., romantic partners, parents, siblings, friends, or fugitive acquaintances) may also be of interest for examining different attachment types (e.g., stable vs. unstable relationships; filial vs. romantic love^5^) and their relevance as social buffers in the face of immediate and/or prolonged periods of threat and stress (e.g.,^60,61^).

Another noteworthy aspect regards the lack of predicted main effects of face category on defensive responding. In a previous study, we observed that viewing loved faces inhibited the defensive startle reflex^5^. However, this was not replicated in the present study. Whereas divergent findings may relate to different tasks (passive viewing vs. instructed threat) and/or reduced trial numbers, other alternative hypotheses are of interest. Specifically, an over-generalization of threat might have occurred across face categories^62^, and/or overwritten the implicit affective picture qualities through verbal instructions^28,33^. This also relates to clinical phenomena, which are observable, for example, in the emergence and treatment of phobias, panic, or trauma-related disorders. While the physical presence of loved ones may help patients to undergo exposure sessions, however, this accompanied exposure could also reinforce fears ‘of not making it alone’. Thus, the present findings do not support the notion that loved ones may act as implicit safety cues, nor evolutionary prepared safety signals.

In summary, this study shows that pictures of loved familiar people readily acquire threatening qualities. The mere verbal instruction about shock threat was sufficient to provoke a pronounced pattern of defensive physiological responding, even when loved ones served as instructed threat cues. Moreover, language information was highly effective to reverse such threat/safety association. Thus, the present data do not support the notion that loved people are per se safe or resistant to becoming threat cues. In contrast, as we know from the clinical domain (e.g., familial abuse and neglect^6^), specifically loved ones can become a source of harm and grief. From a developmental perspective, future research could focus on the accelerating and buffering aspects of interpersonal relationships in modulating (mal-) adaptive social threat and safety learning to cope with adverse life events, sensitive transition periods, and challenging environmental conditions (e.g.^8,63,64^).

## Supplementary Information

Supplementary Information.
