# Supplementary material for: Verbal threat learning does not spare loved ones
Source: Sci Rep. 2021 Mar 9;11:5469. doi: 10.1038/s41598-021-84921-3 (PMC7970900; doi:10.1038/s41598-021-84921-3)

# Supplementary material to the manuscript:

Verbal threat learning does not spare loved ones

Cristina Morato ^1^ Pedro Guerra ^1^, & Florian Bublatzky ^1,2 *^

^1^ Department of Personality, University of Granada, Spain

^2^ Department of Psychosomatic Medicine and Psychotherapy, Central Institute of Mental Health Mannheim, Medical Faculty Mannheim / Heidelberg University, Germany

## Content:

Supplement 1: Assignment of face identities to threat/safety condition

## Supplement 1: Assignment of face identities to threat/safety condition

Because previous research showed that familiarity with loved face pictures can modulate face processing (e.g. Guerra et al., 2011, 2012; Grasso et al., 2009), we aimed at controlling the potential impact of familiarity on our key threat/safety manipulation. To this end, always one parent (i.e. a high-familiar person with whom the participant has lived together for at least 18 years) and one less-familiar person (i.e. partner or best friend with whom the participant was not living together) served as instructed threat/safety cue in the instantiation block. For the reversal block, however, we needed to loosen this restriction in order to have each face identity equally often as a maintained threat/safety and reversed threat-to-safe and safe-to-threat cue. This resulted in eight different combinations that are illustrated in the figure below. Please note, to enable our key comparison between face categories (loved vs. unknown), always the corresponding ‘loved/unknown’ faces of another participant served as threat/safety cues, which led to 4 threat and 4 safety identities in both experimental blocks.


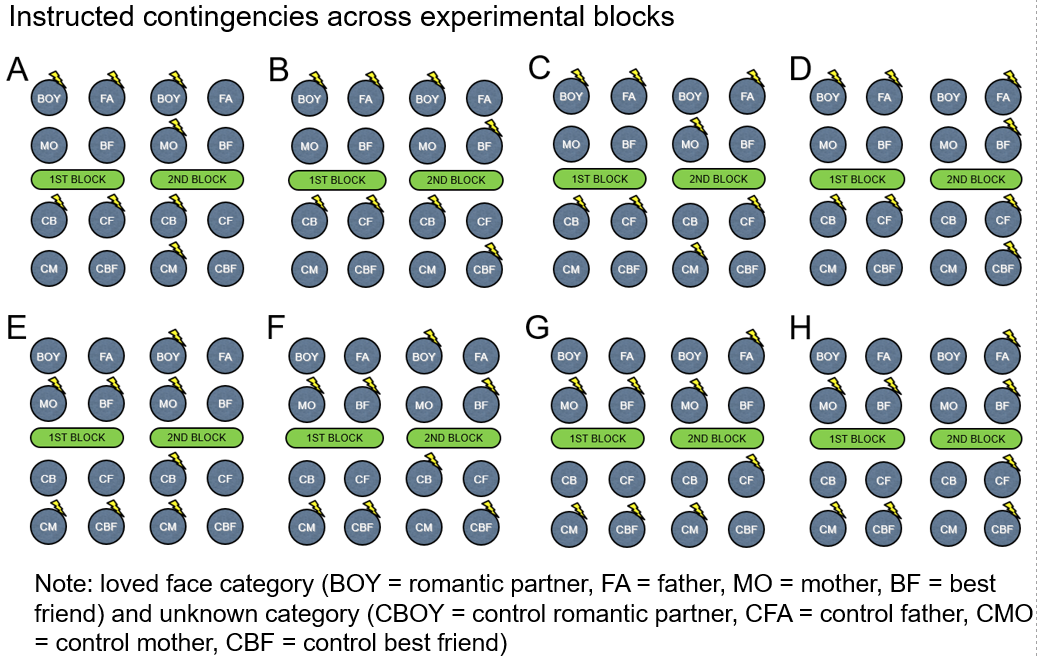

Supplement: Supplementary file 1 — Supplementary Information. [file 41598_2021_84921_MOESM1_ESM.docx]
